# Preoperative radiomic signature based on CT images for noninvasive evaluation of localized nephroblastoma in pediatric patients

**DOI:** 10.3389/fonc.2023.1122210

**Published:** 2023-04-20

**Authors:** Xiao-Hui Ma, Jing Yang, Xuan Jia, Hai-Chun Zhou, Jia-Wei Liang, Yu-Shuang Ding, Qiang Shu, Tianye Niu

**Affiliations:** ^1^ The Children’s Hospital, Zhejiang University School of Medicine, National Clinical Research Center for Child Health, Hangzhou, Zhejiang, China; ^2^ Women’s Hospital, Zhejiang University School of Medicine, Hangzhou, Zhejiang, China; ^3^ Institute of Translational Medicine, Zhejiang University, Hangzhou, Zhejiang, China; ^4^ Institute of Biomedical Engineering, Shenzhen Bay Laboratory, Shenzhen, China; ^5^ Peking University Aerospace School of Clinical Medicine, Aerospace Center Hospital, Beijing, China

**Keywords:** nephroblastoma, radiomics, machine learning, predictive modeling, localized stage

## Abstract

**Background:**

Nephron sparing nephrectomy may not reduce the prognosis of nephroblastoma in the absence of involvement of the renal capsule, sinus vessels, and lymph nodes, However, there is no accurate preoperative noninvasive evaluation method at present.

**Materials and methods:**

105 nephroblastoma patients underwent contrast-enhanced CT scan between 2013 and 2020 in our hospital were retrospectively collected, including 59 cases with localized stage and 46 cases with non-localized stage, and then were divided into training cohort (n= 73) and validation cohort (n= 32) according to the order of CT scanning time. After lesion segmentation and data preprocessing, radiomic features were extracted from each volume of interest. The multi-step procedure including Pearson correlation analysis and sequential forward floating selection was performed to produce radiomic signature. Prediction model was constructed using the radiomic signature and Logistic Regression classifier for predicting the localized nephroblastoma in the training cohort. Finally, the model performance was validated in the validation cohort.

**Results:**

A total of 1652 radiomic features have been extracted, from which TOP 10 features were selected as the radiomic signature. The area under the receiver operating characteristic curve, accuracy, sensitivity and specificity of the prediction model were 0.796, 0.795, 0.732 and 0.875 for the training cohort respectively, and 0.710, 0.719, 0.611 and 0.857 for the validation cohort respectively. The result comparison with prediction models composed of different machine learning classifiers and different parameters also manifest the effectiveness of our radiomic model.

**Conclusion:**

A logistic regression model based on radiomic features extracted from preoperative CT images had good ability to noninvasively predict nephroblastoma without renal capsule, sinus vessel, and lymph node involvement.

## Introduction

Nephroblastoma, also known as Wilms tumor, is the most common renal tumor in pediatric patients (1), accounting for more than 90% of all malignant kidney tumors in children (2), it is also the most frequent pediatric abdominal malignant tumor and the fourth most frequent pediatric malignant tumor overall (3). Under the comprehensive treatment of surgery, chemotherapy, and radiotherapy, the five-year survival rate of nephroblastoma patients has exceeded 90% (4). The current focus of treatment is on how to better protect kidney function and improve long-term survival quality based on ensuring survival rates.

Nephron sparing surgery (NSS) helps to protect the kidneys in the long term and prevent the development of chronic kidney disease, which is especially important in children with a long survival period and facing progressive renal insufficiency (5, 6). Since the successful application in bilateral nephroblastoma, NSS has also been increasingly used to remove selected cases of unilateral nephroblastoma (7–9). Studies have found that in the group of patients with localized disease, the event-free survival and overall survival after NSS appeared to be as good as after total nephrectomy (TN), and local recurrence rates were the same as after TN (8). The application of NSS in unilateral nephroblastoma is limited due to the possible risk of recurrence caused by positive margins. If nephroblastoma patients without the involvement of renal capsule, sinus vessels, and lymph nodes (named localized stage here) can be accurately distinguished before surgery, it will be of great significance to the management of nephroblastoma and will promote the precision treatment of nephroblastoma.

Radiomics is a novel technology that can extract a large number of quantitative information describing the pathophysiological status and phenotypic characteristics of lesions from CT, PET, MRI and other medical images (10–12). Models can be built through in-depth analysis of the extracted mineable radiomic features, combined with clinical examinations and testing information, and using suitable machine learning algorithms to provide clinical decision support. It has shown great advantages in metastasis prediction (13), tumor diagnosis (14), treatment response evaluation (15), and prognosis analysis (16).

The purpose of this study is to establish a radiomic approach based on CT images to develop a non-invasive tool for preoperative prediction of localized nephroblastoma of pediatric patients.

## Materials and methods

### Patients and study flow diagram

This retrospective study was approved and the requirement for informed consent was waived by the Institutional Review Board of our participating institution (Children’s Hospital of Zhejiang University School of Medicine, Zhejiang, China). Inclusion criteria are: (a) computed tomography (CT)-enhanced abdominal scan before surgery, biopsy, radiotherapy, or chemotherapy; (b) successful tumor resection; (c) postoperative pathology proved to be nephroblastoma. Exclusion criteria are: (a) Contrast-enhanced CT imaging of the tumor was not accomplished or treatment was performed before CT scanning; (b) CT images were poor (e.g. motion artifacts); (c) Radiotherapy, chemotherapy or both were applied prior to surgery; (d) Pathology was unclear; (e) bilateral nephroblastoma. We retrospectively collected 105 patients diagnosed as nephroblastoma from December 2013 to September 2020. There are 59 patients in non-localized stage and 46 patients in localized stage. These patients were divided into the training and validation cohorts according to the order of CT scanning time (training cohort, December 2013 to March 2019; validation cohort, April 2019 to September 2020). The training cohort and the validation cohort consist of 73 patients and 32 patients, respectively. Details of our dataset in this study are provided in **Table 1**. The flow diagram of this radiomics study consists of lesion segmentation, data preprocessing, feature extraction, feature selection and model construction. More details are provided below.

**Table 1:** Details of our dataset in this study.

	Training cohort	Validation cohort	Total
**localized**	41	18	59
**Non-localized**	32	14	46
**Total**	73	32	105

### Definition of localized and non-localized stage

Localized stage refers to stage I of the COG nephroblastoma staging system1, excluding items related to surgery.

- Tumor is limited to the kidney.
- The renal capsule is intact.
- The tumor is not ruptured or biopsied before being removed.
- No involvement of renal sinus vessels.
- All lymph nodes sampled are negative.

All the other cases were considered as non-localized stage, equivalent to preoperative stage II-V.

Both localized and non-localized stage were identified based on postoperative pathology.

### CT image acquisition and lesion segmentation

Before CT examination, all of the patients were asked to fast for 4 to 6 hours. Somatom Emotion 16 (SIEMENS) and Optima CT660 CT (GE Medical Systems) were used for examination. Scanning parameters of Somatom Emotion 16 CT scanner were tube voltage 110 kV, tube current 75 mA, layer thickness 1.5 mm, field of view (FOV) 350mm × 350 mm, matrix 512 × 512. Parameters of GE Optima CT660 CT were tube voltage 120 kV, tube current 80 mA, layer thickness 0.625 mm, FOV 350mm × 350 mm, and matrix 512 × 512. High pressure syringe (Mallinckrodt Injection System, Liebel-Flarsheim Co.) was used for nonionic iodine contrast agent injection according to body weight (1.5 ml/kg) at a rate of 1.5-2 ml/s. Portal venous phase images were scanned 50s after the injection.

Portal venous phase CT images were uploaded to a secure laptop. Then 3-Dimensional Slicer (3DSlicer, 4.11.0, http://www.slicer.org/) was used to delineate the three-dimensional (3D) regions of the renal masses with a semi-automatic segmentation procedure, which was performed by two senior radiologists who had more than 10 years of clinical experience. Both of the results they obtained were used to extract radiomic features for evaluating inter- and intra- observer repeatability through Intra Class Correlation Coefficient (ICC).

### Data preprocessing and feature extraction

**Figure 1:**
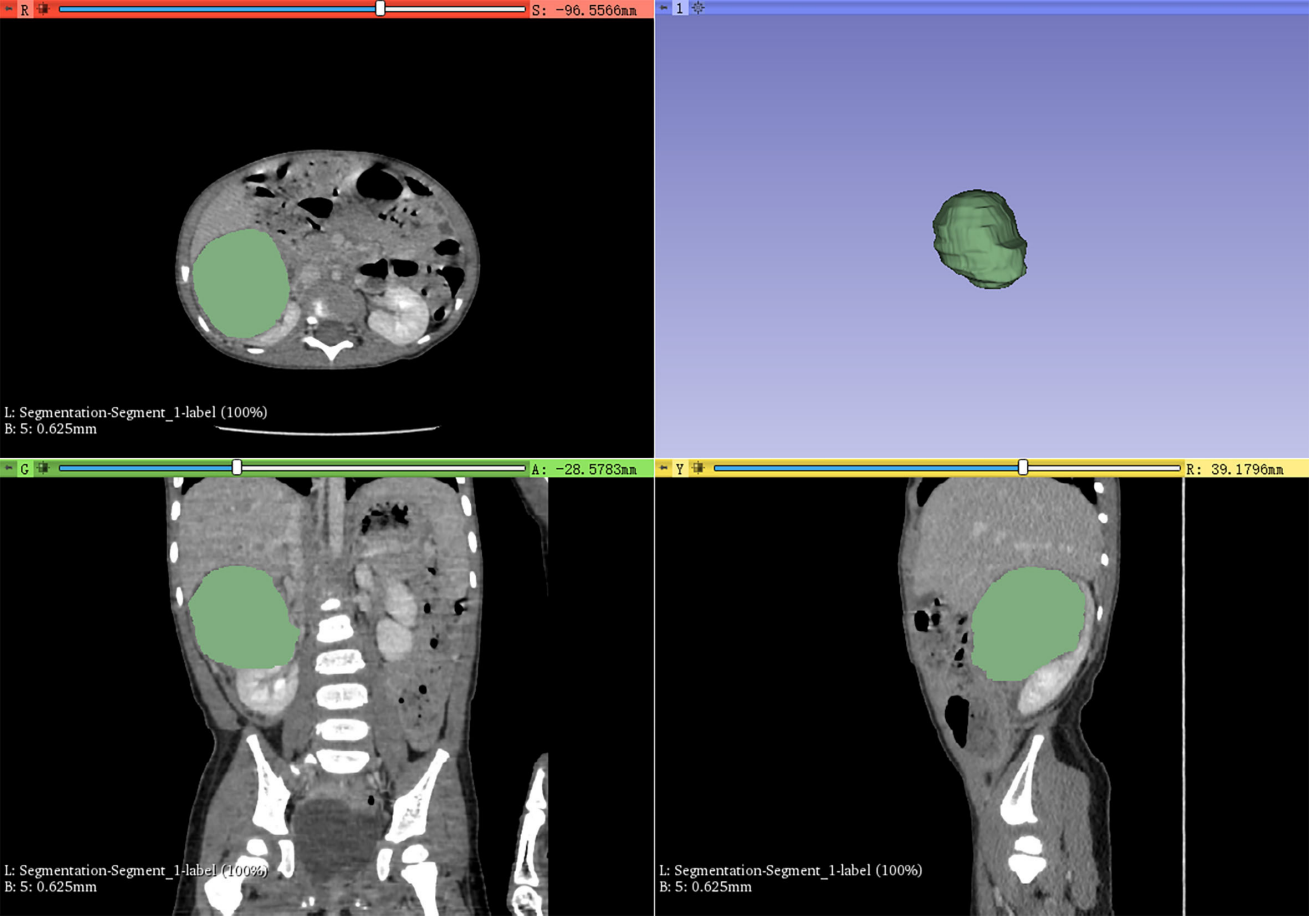
3D-Slicer was used to semi-automatically delineate the whole mass as the volume of interest for radiomics feature extraction.

Volume of interest (VOI) including the whole mass of each nephroblastoma patient was segmented according to radiologists’ delineation (**Figure 1**), then, VOI of all the images were resampled and gray-level normalized by PyRadiomics before feature extracting. We utilized the nearest neighbor algorithm to interpolate each VOI into isotropic data. The voxel spacing of our isotropic data was set to 1×1×1 mm^3^, 3×3×3 mm^3^, or 5×5×5 mm^3^.

Radiomic features were extracted from original and derived interpolated VOIs using PyRadiomics v2.2.0 (17), an open-source python package (https://pyradiomics.readthedocs.io/en/latest/). These derived interpolated VOIs were obtained by applying six built-in optional filters (including Laplacian of Gaussian, Wavelet, Square, Square Root, Logarithm and Exponential) to original interpolated VOIs. Feature classes consist of shape, first order statistics and texture features. Texture features contain gray level cooccurence matrix (GLCM), gray level run length matrix (GLRLM), gray level size zone matrix (GLSZM), neigbouring gray tone difference matrix (NGTDM), and gray level dependence matrix (GLDM) features. All radiomic features were normalized (Z-score) to balance the feature contribution and make each feature at the same quantity level. All feature classes with the exception of shape can be extracted on original and derived interpolated VOIs.

### Feature selection and model construction

In this study, we devised a multi-step procedure for the selection of radiomic features to produce radiomic signature. First, Pearson correlation analysis (13, 18, 19) was used to identify the redundancy of radiomic features in the training cohort. We calculated Pearson correlation coefficients of pair-wise radiomic features to build Pearson correlation matrix. Each feature with the mean absolute correlation higher than Pearson threshold was considered redundant thus eliminated. The Pearson threshold between 0.70 and 0.95 (the stride was 0.5) was utilized to identify the highly correlated feature pairs. Then the radiomic signature was identified by sequential forward floating selection (SFFS) algorithm (20). We utilized Logistic Regression (LR), ten-fold cross validation, and area under the receiver operating characteristic (ROC) curve (AUC) value as the classifier, K-fold cross validation, scoring criterion of the SFFS algorithm respectively. The number of retained features varies from 1/15 to 1/10 of total patient sample size (the stride was 1) (21–23), that is, the number of retained radiomic features ranged from 7 to 11. Next, the corresponding radiomic signature of the validation cohort were obtained according to the radiomic signature of the training cohort.

**Figure 2:**
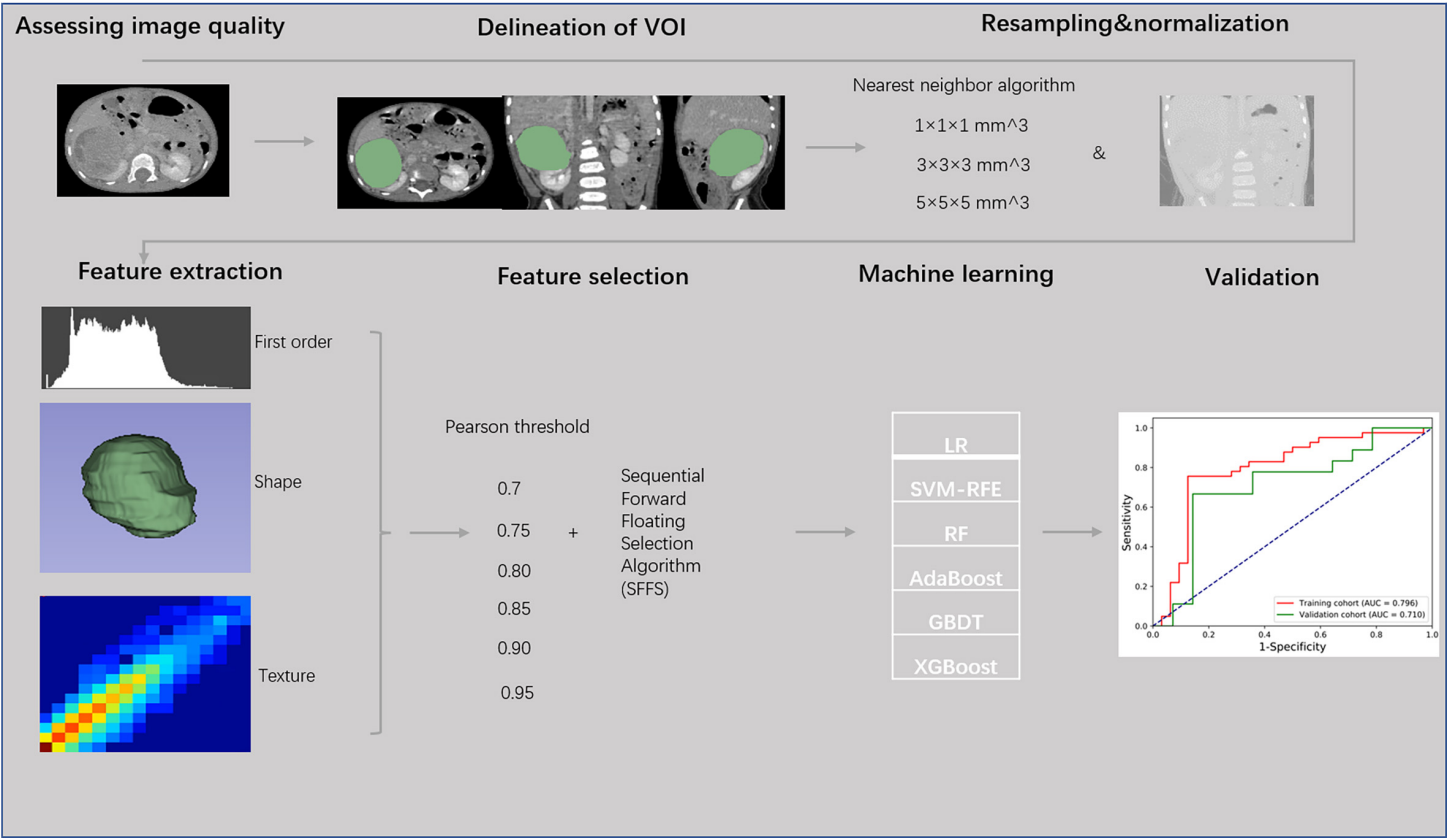
Flow chart of radiomics analysis of localized nephroblastoma.

Considering the voxel spacing of isotropic data, the Pearson threshold and the number of retained features vary the radiomic signature, we constructed prediction models with different parameters and LR classifier to classify patients with localized and non-localized nephroblastoma in the training cohort. The discrimination performance was quantified by the AUC value. In addition to the LR classifier, we further compared the performances of prediction models using the optimal parameter combination and other machine learning classifiers. These classifiers include Support Vector Machine with Recursive Feature Elimination (SVM-RFE), Random Forest (RF), Adaptive Boosting (AdaBoost), Gradient Boosting Decision Tree (GBDT), and eXtreme Gradient Boosting (XGBoost). Finally, the performance was validated in the validation cohort. The flow chart was shown in **Figure 2**.

## Results

A total of 105 patients (50 males/55 females) were enrolled in the study, including 59 cases with non-localized stage and 46 cases with localized stage. They were divided into training cohort (n= 73, 37 males/36 females) and validation cohort (n= 32, 13 males/19 females) according to the order of CT scanning time. Age of the total study sample ranged from 0.1 to 10.3 years, with a median age of 2.1 years. Age of the training cohort ranged from 0.1 to 7.9 years with a median age of 1.9 years, whereas age of the validation cohort ranged from 0.1 to 10.3 years with a median age of 2.9 years.

A total of 1652 radiomic features have been extracted on each VOI. For original interpolated VOIs, 105 radiomic features were obtained including 14 shape features, 18 first order statistics features and 73 texture features. The remaining 1547 radiomic features were extracted from derived interpolated VOIs. They consisted of 306 first order statistics features and 1241 texture features. ICC analysis showed that the radiomic features from the VOIs delineated by the two senior radiologists had good inter- and intra- observer repeatability (ICC ranged from 0.73 to 1.0).

**Figure 3:**
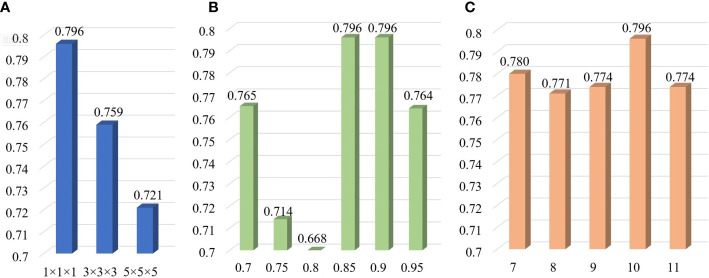
AUC values of LR models with various parameters including the voxel spacing of isotropic data **(A)**, the Pearson threshold **(B)** and the number of retained features **(C)** in the training cohort.

In order to determine the optimal parameter combination, we have adjusted different parameters (including the voxel spacing of isotropic data, the Pearson threshold and the number of retained features) and then established the corresponding LR models in the training cohort. The LR model with the optimal parameter combination was chosen as the final LR model. We found that the optimal parameter combination was the voxel spacing of 1×1×1 mm^3^, the Pearson threshold of 0.90 and the feature number of 10. When one parameter remains unchanged and other parameters change, the AUC values of the LR models are shown in **Figure 3**. The AUC value decreases with the increase of the voxel spacing of isotropic data, illustrating that clearer image data is more conducive to radiomics analysis. A total of 11 patients (including 4 cases of non-localized stage and 7 cases of localized stage) were able to correctly predict whether they were localized or not, even if the voxel spacing was changed. As for the Pearson threshold and the number of retained features, no clear pattern was observed in this single-center study. In the Pearson correlation analysis, a total of 354 radiomic features (including 3 shape features, 73 first order statistics features and 278 texture features) were obtained with threshold 0.90. About 78.57% of radiomic features have been eliminated. After the SFFS feature-ranking algorithm, top 10 radiomic features (including 3 first order statistics features and 7 texture features) were selected as our radiomic signature (**Table 2**).

**Table 2:** Details of the radiomic signature in this study.

Feature	Image Type	Feature Class	Specific Name
**Feature_1**	Log-sigma-5-0-mm-3D	NGTDM	Strength
**Feature_2**	Wavelet-HLH	GLSZM	LargeAreaLowGrayLevelEmphasis
**Feature_3**	Wavelet-HHH	GLCM	ClusterShade
**Feature_4**	Wavelet-HHH	GLSZM	GrayLevelNonUniformity
**Feature_5**	Wavelet-LLL	Firstorder	Maximum
**Feature_6**	Square	Firstorder	Kurtosis
**Feature_7**	Square	GLSZM	SmallAreaEmphasis
**Feature_8**	Square	GLSZM	ZoneVariance
**Feature_9**	Logarithm	GLCM	ClusterProminence
**Feature_10**	Exponential	Firstorder	Kurtosis

“Log-sigma-5-0-mm-3D” means that the 3D Laplacian of Gaussian filter with the kernel width “sigma” of 5.0 was implemented. “Wavelet-HLH” means that a high-pass filter, a low-pass filter and a high-pass filter was applied on x, y and z-axis of the wavelet filter, which was named analogously for “Wavelet-HHH” and “Wavelet-LLL”. “Firstorder” means first order statistics.

In addition to the final LR model, SVM-RFE, RF, AdaBoost, GBDT, and XGBoost models were constructed using the optimal parameter combination and the corresponding machine learning classifiers. **Table 3** shows the AUC values of these models for predicting the localized status in patients with nephroblastoma. Although the same radiomic signature was used, only AUC values of the final LR model were higher than 0.70 in the training and validation cohorts (training cohort, 0.796; validation cohort, 0.710). Other prediction models performed poorly in distinguishing localized group from non-localized group. It designates the potential of LR-based radiomics in classifying localized and non-localized nephroblastoma in pediatric patients. For the final LR model, the radiomic signature calculation formula is presented as follows: Rad_ signature = 0.22 - 0.52 × Feature_1 - 0.56 × Feature_2 - 0.21 × Feature_3 - 0.35 × Feature_4 - 0.84 × Feature_5 - 0.02 × Feature_6 + 0.58 × Feature_7 + 0.27 × Feature_8 + 0.33 × Feature_9 - 0.31 × Feature_10.

**Table 3:** AUC values of different prediction models for predicting the localized nephroblastoma.

Model	Training cohort	Validation cohort
LR	0.796	0.710
SVM-RFE	0.697	0.808
RF	0.643	0.657
AdaBoost	0.531	0.619
GBDT	0.608	0.647
XGBoost	0.643	0.694

**Figure 4:**
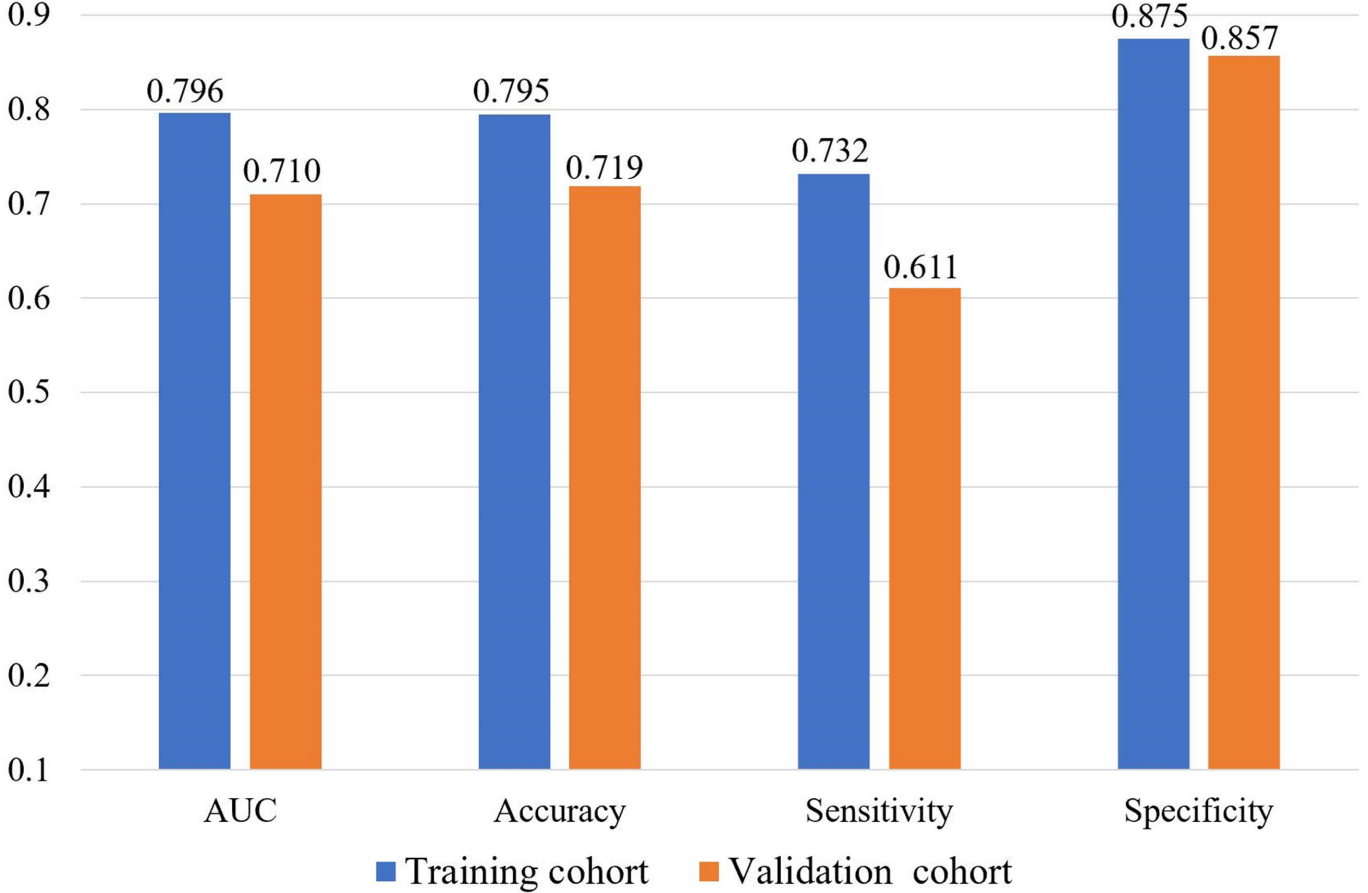
Performances of the final LR model for predicting the localized nephroblastoma.

**Figure 5:**
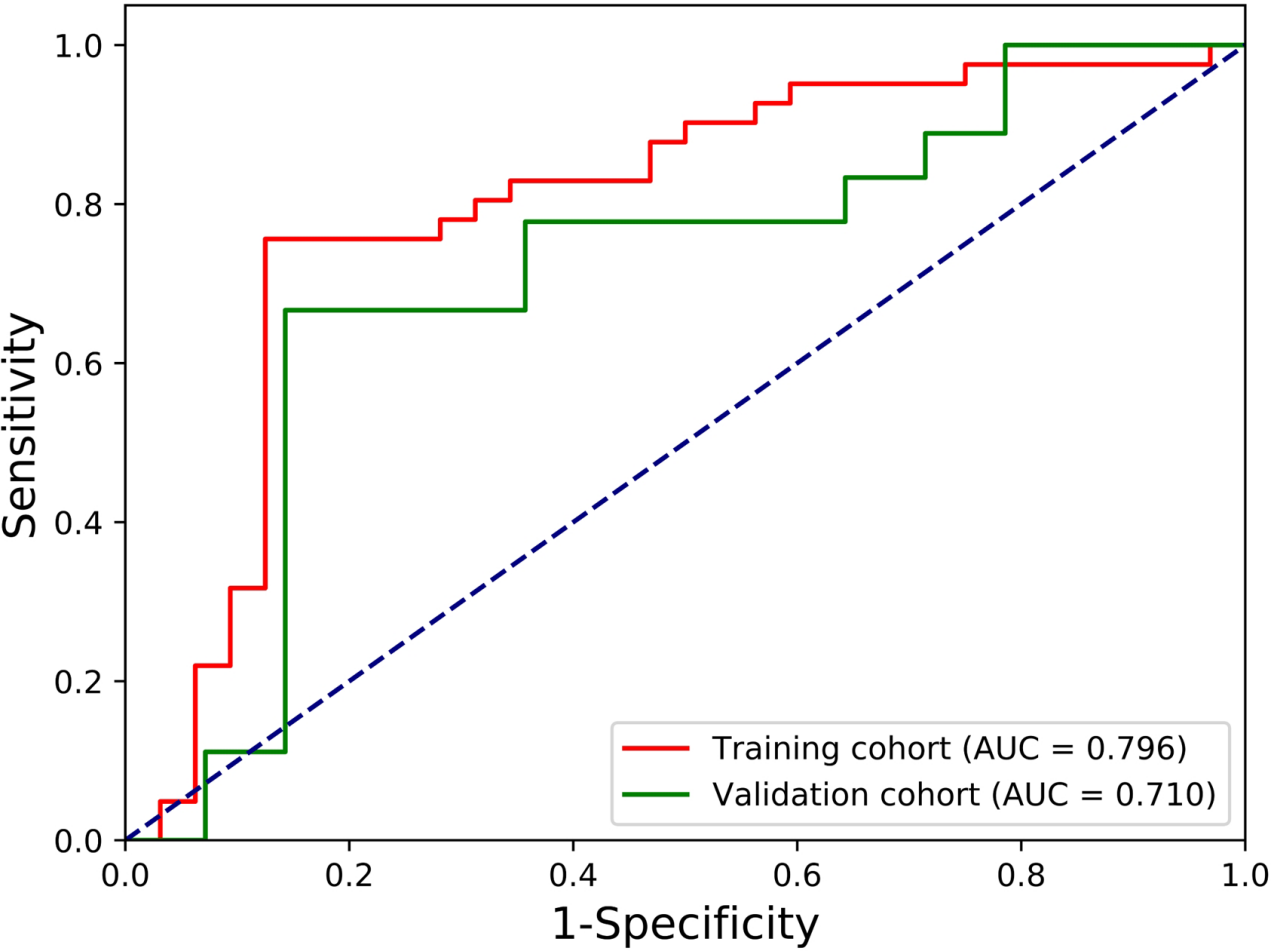
ROC curves of the final LR model for predicting the localized nephroblastoma.

We further explored the performances of our radiomic signature calculation formula based on the final LR model using more performance metrics (such as accuracy, sensitivity and specificity). All performance metrics of the final LR model for predicting the localized nephroblastoma are presented in **Figure 4**. The AUC, accuracy, sensitivity and specificity are 0.796, 0.795, 0.732 and 0.875 for the training cohort respectively, and 0.710, 0.719, 0.611 and 0.857 for the validation cohort respectively. Results manifest the effectiveness in distinguishing patients with localized and non-localized nephroblastoma (**Figure 5**).

## Discussion

The overall survival outcomes of most nephroblastoma patients are good, and further studies should focus on how to use the optimal treatment under more precise risk-stratified strategies (24) to improve the long-term prognosis of patients with nephroblastoma. Our result can be regarded as a kind of non-invasive risk-stratified strategies prior to surgery for nephroblastoma patients using radiomics technique, which goes beyond the ability of the human eye to identify the completely localized status in CT images.

Unlike previous surveys that concerned of all stages of nephroblastoma, our current study highlighted on the detection of nephroblastoma patients at a very early stage without involvement of renal capsule, sinus vessels, and lymph nodes. These patients can not only undergo complete tumor resection, but also have the potential to perform nephron sparing nephrectomy. Therefore, we developed a new method to divide patients into localized group and non-localized group non-invasively before operation, so as to guide the personalized treatment of nephroblastoma. To the best of our knowledge, the relationship between radiomic features and the involvement of renal capsule, sinus vessels, and lymph nodes of nephroblastoma has not been evaluated previously.

At present, lots of studies have shown that radiomic technology has the power to predict metastasis of different diseases. For example, one of our previous studies showed that radiomic features could effectively predict preoperative lymph node metastasis in patients with gastric cancer (13). In that study, tumor radiomics and LN radiomics were integrated to build an effective non-invasive tool to guide treatment decision-making in gastric cancer, which was notably useful in patients with T2-stage, diffuse subtype, and moderately/well differentiated gastric cancer. Zhao et al. found that radiomic features from preoperative CT images could predict future distant metastasis of local renal cell carcinoma after surgical resection, and these predictive radiomic features were related to certain important biological pathways (extracellular matrix-receptor interaction, focal adhesion and Phosphoinositide 3-kinases/Akt pathways) (25). Their study suggested that these findings provided support for the biological interpretation of radiomics model and might enhance the molecular basis of radiomics. Both of the above studies showed that the radiomic technology has offered a possible solution for studying the correlation between medical imaging and tumor metastasis in patients with nephroblastoma.

In this study, we found that the radiomic features extracted from enhanced CT images of preoperative nephroblastoma patients could be used as an independent predictor of localized nephroblastoma. 10 radiomic features related to early localized stage of nephroblastoma were screened out from a large amount of data through a generalized methodology for radiomic feature selection and modeling developed by our team (23), in which Pearson correlation analysis and SFFS algorithm were used for feature selection. The methodology was verified by our own three data sets (Gastric cancer dataset, Osteosarcoma dataset, Pancreatic neuroendocrine tumors dataset) and performed well in these three different origin and different types of solid tumors, which proves the stability and reliability of this methodology in feature selection. Similarly, the results of our LR-based model in this study demonstrated the effectiveness of radiomic features based on preoperative enhanced CT images for predicting localized nephroblastoma at the training cohort (AUC, 0.796; accuracy, 0.795; sensitivity, 0.732; specificity, 0.875) and the validation cohort (AUC, 0.710; accuracy, 0.719; sensitivity, 0.611; specificity, 0.857).

In addition to a single machine learning classifier, the performance of different classifiers based on same radiomic features was also worth exploring. Parmar et al. found that classifiers had a significant impact on the model performance (accounting for 34.21% of the total model differences) (26), so it was a very important step to determine the best machine learning classifier and apply it to radiomics. Our results also supported this conclusion. Through the comparison of 6 machine learning classifiers (LR, SVM-RFE, RF, AdaBoost, GBDT, and XGBoost), we found that LR was the most suitable classifier for our dataset –only the AUC of LR was higher than 0.75 in training cohort (training cohort: 0.796), and it performed equally well on the validation cohort (validation cohort: 0.710). Dong et al. applied multiple machine learning methods based on radiomics to differentiate intracranial ependymoma from medulloblastoma and found that the performance of RF classifier was the best (AUC 0.91,95% CI 0.787-0.968), followed by Support Vector Machines and K neighbors, and Adboost was the worst (AUC 0.75,95% CI 0.604-0.857) (27). Then Paired test showed significant difference (P<0.05) in ROC curve among the classifiers used. In this study, we also compared the model performance of LR with other machine learning methods, which used the same radiomic features for predicting the absence of involvement of the renal capsule, sinus vessels, and lymph nodes in nephroblastoma. The results illustrated that the radiomic model of LR achieved the best performance both in the training cohort and validation cohort (P<0.05).

By comparing radiomic features based on three different types of interpolated images, it was found that features extracted from images with the smallest voxel spacing (1 × 1 × 1 mm^3^) resulted in the best model accuracy, which was consistent with the truth that smaller voxel associated with the higher resolution (28), even though this smaller voxel was obtained by a resampling technique.

Despite the aforementioned important findings, our study still had several limitations. First of all, this study was retrospective and had a relatively small size of cohort, although external validation was used. In order to obtain a higher level of clinical application evidence, it needed to be verified with prospective and larger data sets. Secondly, our study did not take into account some other features, such as clinical features, genomics and so on. The combination of multi-dimensional data may further improve the predictive ability of the model and thus providing better clinical decision support.

Overall, the results showed that the LR-based radiomic model had a good ability of non-invasive prediction of nephroblastoma without renal capsule, sinus vessels and lymph node involvement., and had the potential to help make surgical decisions for precise treatment and improve the long-term quality of life of nephroblastoma patients. However, more data is needed to improve the global applicability and robustness of the method.

## Data availability statement

The original contributions presented in the study are included in the article/supplementary material. Further inquiries can be directed to the corresponding authors.

## Ethics statement

The study was conducted in accordance with the Declaration of Helsinki (as revised in 2013). The study was approved by the ethics committee of the Children’s Hospital, Zhejiang University School of Medicine (2021-IRB-097) and individual consent for this retrospective analysis was waived.

## Author contributions

X-HM and JY designed this project and took charge of the development of machine learning and writing this paper. H-CZ and XJ delineated the area of interest in our medical images. J-WL and Y-SD had equal contributions in preparing figures and clinical information. TN and QS supervised the development of this project and guided the writing of this article. X-HM and JY takes mainly responsibility for the integrity of the content of the paper and each author have participated sufficiently in the submission to take public responsibility for the content. All authors contributed to the article and approved the submitted version.
